# External Guidewire Direction as an Adjunctive Clue for Accurate Central Venous Catheter Placement: A Case Report

**DOI:** 10.7759/cureus.85736

**Published:** 2025-06-10

**Authors:** Azeez M Aspari, Neingutso Lomi, Anirban Bhattacharjee, Habib Md R Karim, Bheemas Atlapure

**Affiliations:** 1 Anaesthesiology, Critical Care, and Pain Medicine, All India Institute of Medical Sciences, Guwahati, Changsari, IND

**Keywords:** central venous access, cvc malposition management, deranged coagulopathy, potential diagnostic aid, ultrasound-guided vascular access

## Abstract

Central venous catheterization (CVC) under ultrasound guidance is a widely adopted and current standard practice that enhances safety and accuracy, particularly in high-risk patients. As the procedure provides a visual aid, we usually expect fewer complications. We report a case of right internal jugular vein (IJV) central catheter placement where the CVC guidewire was misplaced after IJV puncture under ultrasound guidance. A 65-year-old male with advanced liver failure and coagulopathy underwent CVC placement. The direction of the external guidewire segment suggested some abnormalities. Repeat ultrasonographic confirmation, following up on the guidewire, identified an internal misplacement; the guidewire had pierced the medial wall of the IJV and crossed the midline. Real-time ultrasound was then used to guide the repositioning of the guidewire and the subsequent correct placement of the CVC. Real-time ultrasound guidance for repositioning prevented repeat puncture, which increases the risk of bleeding, especially in high-risk patients. This case highlights the value of following up guidewire placement beyond the puncture point entry as a routine procedure, emphasizing the critical role of visualizing the guidewire entry into the brachiocephalic vein as confirmatory. Further, it highlights that external guidewire segment orientation is a valuable adjunct to ultrasound guidance during CVC placement, especially in patients with complex anatomy.

## Introduction

Central venous catheterization (CVC) is frequently required while caring for critically and acutely ill patients and patients undergoing chemotherapy, hemodialysis, and major surgeries. The procedure is considered safe, primarily when performed under ultrasound guidance. Current practice has widely adopted ultrasound-guided placements as a standard for such line placements, enhancing safety and accuracy, particularly in high-risk patients [1,2]. Liver failure is frequently associated with coagulopathies due to poor clotting factor production and low platelet counts. Although coagulopathy and low platelet counts are regarded as possible risks for bleeding for CVC, the incidence of significant bleeding is minimal, especially with the use of ultrasound-guidance. Thus, CVC placement, even in patients with liver failure with coagulopathy, is considered relatively safe [3].

Nevertheless, more than one venous puncture and failure to pass the guidewire are risks for minor bleeding and hematoma [3]. Ultrasonographic real-time procedure is likely to reduce such complications [4]. Although internal guidewire positioning is typically confirmed using ultrasonography, a gap remains in confirming the guidewire's position within the vein by following up, and misplacement can still occur, even with ultrasonographic assistance, especially when the evaluation is limited to the entry point only. In such a scenario, an adjunctive indicator might be helpful for increased vigilance. We present such an adjunctive indicator; the direction of the external guidewire can provide additional practical clues to assist clinicians in the early detection of malpositioning.

## Case presentation

A 65-year-old male patient of average build, poorly nourished, with advanced liver failure, massive ascites, and a deranged coagulation profile, required central venous access via the right internal jugular vein (IJV) for clinical management. He was conscious, alert, oriented, and calm. The cardio-respiratory system was grossly standard without apparent signs of failure and distress. Mild icterus and bilateral mild pitting edema were notable. His laboratory report pertinent to liver function and coagulation profile is presented in Table 1. Child-Turcotte-Pugh Classification for severity classified the patient as class C (severe disease).

**Table 1:** Liver function and coagulation test results with reference levels.

Test	Patient Value	Normal Reference Range
Total bilirubin	4.2 mg/dL	0.1 – 1.2 mg/dL
Direct bilirubin	2.8 mg/dL	0.0 – 0.3 mg/dL
Indirect bilirubin	1.4 mg/dL	0.2 – 0.8 mg/dL
Aspartate aminotransferase	112 U/L	10 – 40 U/L
Alanine aminotransferase	85 U/L	7 – 56 U/L
Alkaline phosphatase	230 U/L	44 – 147 U/L
Serum albumin	2.3 g/dL	3.5 – 5.0 g/dL
Prothrombin time	23 seconds	11 – 15 seconds
International normalized ratio	1.8	0.8 – 1.2
Activated partial thromboplastin	44 seconds	25 – 35 seconds
Serum creatinine	1.2 mg/dL	0.6 – 1.2 mg/dL
Platelet count	90000/dL	150,000 – 450,000/dL

Owing to the high risk of bleeding, the procedure was performed under real-time ultrasound guidance using a high-frequency linear transducer (13-6 MHz) with a point-of-care ultrasound system (Sonosite M-Turbo®; FUJIFILM Sonosite Inc., Bothell, Washington, United States). The patient was positioned supine with a mild head rotation to the left to facilitate access to the IJV. The Seldinger technique uses a thin-walled introducer needle and a flexible J-tip guidewire. The procedure began with a short-axis sonographic view to identify and puncture the right IJV. The guidewire was visualized by entering the vein during the initial puncture under real-time ultrasound guidance. A free flow of dark red blood was noted, and no resistance was encountered during the guidewire insertion, giving an initial impression of an uneventful guidewire placement.

**Figure 1:**
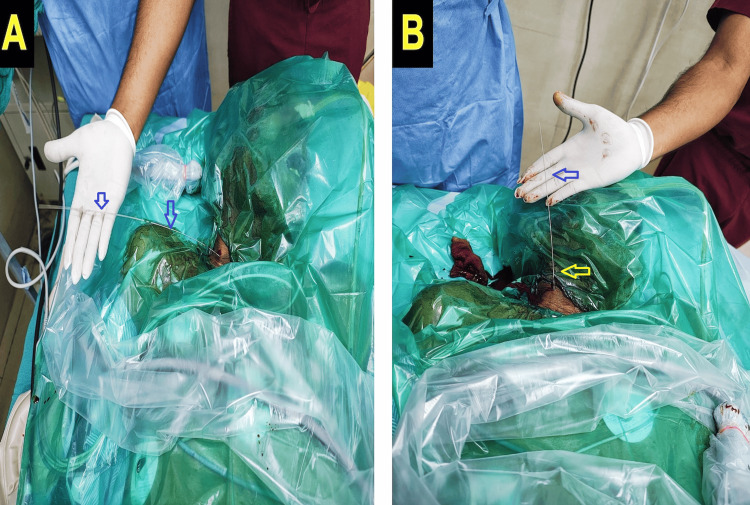
Laterally directed external portion of the guidewire pointing to the right (same side) shoulder for malposition (A), aligned cephalad along the midline when properly placed after repositioning (B).

However, the external portion of the guidewire deviated laterally toward the right shoulder (Figure 1A), raising the suspicion of an abnormal intravascular trajectory. For further evaluation, point-of-care ultrasonography (POCUS) was used to trace the internal course of the guidewire in both short and long axes. The guidewire pierced the medial wall of the IJV and crossed the midline toward the left side, indicating suboptimal positioning and vessel wall perforation. Instead of pulling out the guidewire completely and performing the procedure from scratch, under continuous ultrasound visualization, the guidewire was carefully withdrawn and redirected into the right brachiocephalic vein and the superior vena cava (SVC). Following repositioning, the external portion of the guidewire was found aligned cephalad along the midline (Figure 1B), and the correct course was again confirmed using POCUS (Video 1).

**Video 1:** POCUS video loops of guidewire position. The initial 10 seconds show the guidewire pierce the medial wall of the right IJV; the middle 10 seconds show real-time withdrawal and repositioning of the guidewire (the J-tip of the guidewire is being seen re-entering the lumen). The last portion shows that the guidewire was visualized following the expected intravascular trajectory into the right brachiocephalic vein and SVC, confirming optimal placements. POCUS: point-of-care ultrasound; IJV: internal jugular vein; SVC: superior vena cava

A central venous catheter was subsequently placed without complications, and the patient tolerated the procedure well. Follow-up for the next 48 hours did not show any CVC-related complications.

## Discussion

By this time, ultrasound guidance had become the gold standard for CVC placement. It reduced complications, improved first-pass success, and minimized mechanical injuries such as arterial puncture and pneumothorax [1,2]. Our case highlights two important aspects, namely, the need to close the gap in practice by extending ultrasonographic evaluation to confirm that the guidewire is correctly in situ and using the external guidewire direction as an adjunct to detect suspected guidewire malposition.

The 2020 American Society of Anesthesiologists (ASA) Practice Guidelines strongly recommend real-time ultrasound guidance for vessel localization and venipuncture, particularly when the IJV is selected for cannulation [5]. Static ultrasound imaging is also advocated before prepping and draping to evaluate the vessel anatomy and patency, which enhances procedural planning and safety [5]. Despite ultrasound's benefits, guidewire misplacement can still occur, especially in critically ill patients with altered anatomy, massive ascites, or elevated intra-abdominal pressure, as in the present case [4,6]. Here, although the initial real-time ultrasound confirmed venous puncture, the guidewire passed out of the IJV shortly after entering the vein. This led to the formation of tension in the guidewire, and the external guidewire segment was observed to deviate laterally toward the right shoulder. This atypical external orientation prompted further POCUS evaluation, filling the practice gap and aiding the diagnosis.

Our POCUS finding of the guidewire piercing the medial wall and crossing the midline, which was not immediately apparent during initial cannulation, underscores a vital pitfall: confirmation of vessel entry alone does not guarantee safe intraluminal guidewire passage. The ASA guidelines emphasize that venous confirmation of the guidewire residence is crucial before inserting a dilator or catheter after needle insertion. It can be accomplished using ultrasound, pressure waveform analysis, manometry, or transesophageal echocardiography [5].

Importantly, visualization of the guidewire solely at the entry point is no longer sufficient during real-time ultrasound guidance. The guidewire should be followed distally through the right brachiocephalic vein and into the SVC to ensure accurate placement. It may prevent serious complications, such as double-wall puncture, false passage, and intrathoracic misdirection, and even diagnose them immediately if they occur. This principle is now incorporated into the recent ASA’s algorithm for central venous insertion and verification, which explicitly calls for confirmation of the vein's guidewire position before dilation or catheterization, particularly when using the thin-walled needle (Seldinger) technique [5].

The present case also supports the adjunctive utility of external guidewire direction. A cephalad and midline orientation generally suggests correct progression toward the SVC, while lateral, downward, or erratic trajectories may indicate vessel wall perforation or misplacement [7,8]. These external visual cues, although often overlooked, can be integrated into routine procedural assessment, especially when sonographic windows are limited due to obesity, ascites, or neck deformities. However, such alignment may also be affected if the guidewire gets coiled within the IJV and produces tension within that segment. 

The Third Sonography Outcomes Assessment Program (SOAP-3) trial and multiple systematic reviews have emphasized that combining real-time ultrasound guidance with procedural vigilance, including tactile feedback and external guidewire inspection, can significantly reduce adverse events such as catheter malposition, hematoma, or vessel injury [8,9]. In high-risk patients with anatomical distortions or coagulopathy (e.g., cirrhosis and ascites), a multimodal verification strategy is essential. It includes pre-puncture static ultrasound for vessel identification, real-time ultrasound for cannulation, post-puncture tracking of the guidewire into the brachiocephalic vein and SVC, assessment of external wire orientation, tactile feedback during guidewire advancement, and post-procedural verification of catheter tip position and guidewire retrieval. The ASA guidelines strongly endorse these layered safety checks, which caution against relying solely on visual blood return or non-pulsatile flow to confirm venous access [5].

An audit of the ultrasound-guided CVC placement in adults with liver disease has shown that the procedure is safe even in patients with coagulopathy [10]. The audit evaluated 699 procedures in liver disease patients, where 40% were severe liver disease with a Child C status, and found no vascular and non-vascular complications. However, a backup for such complication management and the need for fresh frozen plasma or other blood components cannot be overstated, and such facilities should also be considered. 

## Conclusions

The present case highlights the integration of multiple safety checks, including real-time and static ultrasound imaging, post-puncture confirmation of guidewire trajectory into the central veins, and observation of external guidewire direction, as a robust and reliable strategy for minimizing complications and early detection during CVC placement in high-risk patients. A comprehensive, dynamic approach is essential for procedural success and patient safety. External guidewire segment orientation might serve as a clue for guidewire misplacement by piercing the medial wall of the jugular vein and thus hinting at the need for re-confirmation of the position. However, our suggestion is based on a single case observation, which needs to be validated by observation in other similar cases in the future. 
